# Zygomatic air cell defect: A panoramic radiographic study of a south Indian population

**DOI:** 10.4103/0971-3026.63052

**Published:** 2010-05

**Authors:** Srikanth HS, Karthikeya Patil, Mahima VG

**Affiliations:** Department of Oral Medicine & Radiology, J. S. S. Dental College & Hospital, Mysore, Karnataka - 570 015, India

**Keywords:** Articular tubercle, pneumatized articular eminence, zygomatic air cell defect

## Abstract

**Aims:**

To determine the prevalence, patterns of occurrence and variations of zygomatic air cell defects (ZACDs) using panoramic radiographs.

**Methods and Materials:**

Dental panoramic radiographs of 600 outpatients were examined to evaluate the variations and characteristics of ZACDs.

**Results:**

ZACDs were identified in 15 subjects out of 600, giving an overall prevalence of 2.5%. Seven ZACDs were seen in males and eight in females. Among the 15 ZACDs, nine were unilateral and six were bilateral.

**Conclusion:**

The overall prevalence of ZACD is relatively low in south Indian population and careful radiographic evaluation is needed to detect these entities.

## Introduction

Pneumatized air cells present in the zygomatic process of the temporal bone are termed Zygomatic Air Cell Defects (ZACDs).[1] ZACDs have been defined as ‘accessory air cells in the zygomatic process and articular eminence of the temporal bone, which appear similar to mastoid air cells and which do not extend further anteriorly than the zygomatico-temporal suture.’[2] This terminology was coined by Tyndall and Matteson in the year 1987; the same investigators had previously named this entity as pneumatized articular eminence.[1,3] In short, ZACDs are nothing but extensions of the mastoid air cells anteriorly into the zygomatic process of the temporal bone.

A classification of ZACDs based on their panoramic radiographic appearance was proposed by Tyndall and Matteson in the year 1985 and has been further used by Carter and coworkers as[4]:

1. Unilocular type
2. Multilocular type
3. Trabecular type

A unilocular ZACD appears as radiolucency with well-defined borders, while the multilocular type demonstrates numerous small cavities within, which resemble mastoid air cells. The trabecular variety is basically a multilocular entity with internal bony striations.[2,4]

When ZACDs have been demonstrated preoperatively on a radiograph, these may become contraindications to performing surgical procedures such as eminoplasty or eminectomy for the treatment of mandibular dislocations as they can become potential pathways for intracranial infections.[4]

## Subjects and Methods

Dental outpatients, in the age range of 20–50 years, visiting the Department of Oral Medicine and Radiology in our institution, were selected by stratified random sampling. The study population comprised 600 subjects divided into three groups of 200 each, according to age; each group consisted of 100 male and 100 female subjects. Group I consisted of subjects in the age range of 20–29 years, group II in the age range of 30–39 years, and group III in the age range of 40–49 years.

Subjects with developmental malformations of the face and jaws, those in whom systemic conditions had affected growth, those with clinical or radiographic evidence of pathologies in the maxillofacial region, and those with a history of trauma to the maxillofacial region and who had been treated with surgical intervention were excluded from the study. Panoramic radiographs of the subjects were made using a panoramic unit (Orthoslice 1000 C, Trophy, France) and were interpreted. The ZACDs were identified and grouped as unilocular and multilocular types and as unilateral or bilateral. The data were tabulated and subjected to statistical analysis.

## Results

The mean age of the study subjects was 34.12 years; 34.23 years in males and 34 years in females. Out of 600 study subjects, ZACDs were found in 15, giving an overall prevalence of 2.5%. The mean age of subjects with ZACDs was 30.2 years. Out of 15 ZACDs, nine were in group I, two in group II, and four in group III. Out of 15 ZACDS, seven were in males and eight were in females. The male to female ratio was 1: 1.17.

**Figure 1:**
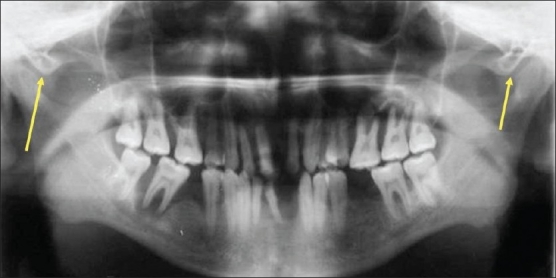
Panoramic radiograph shows bilateral multilocular ZACDs (arrows)

**Figure 2:**
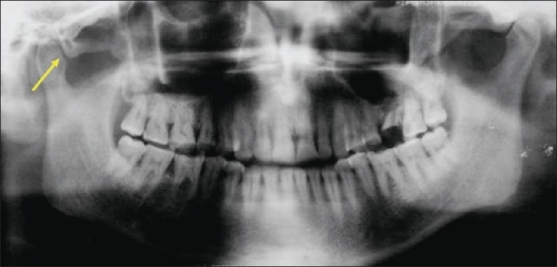
Panoramic radiograph shows a unilateral (R) multilocular ZACD (arrow)

**Figure 3:**
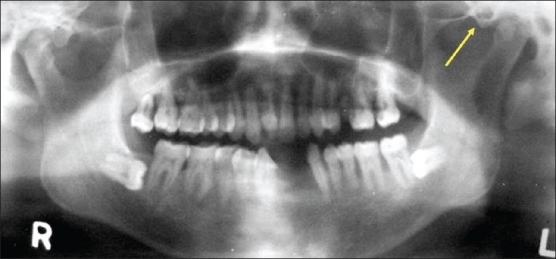
Panoramic radiograph shows a unilateral (L) multilocular ZACD (arrow)

**Figure 4:**
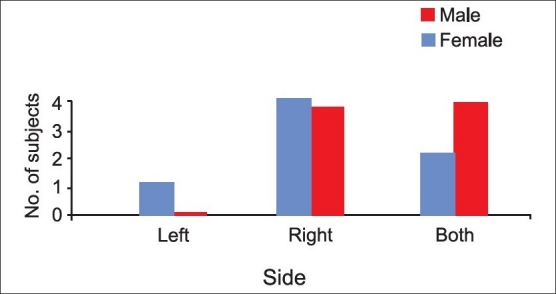
Distribution of ZACDs with respect to side and gender

The mean age of males with ZACD was 32.71 years and it was 28 years in females. Among 15 ZACDs, nine were unilateral and six were bilateral. Among the nine unilateral ZACDs, five were seen in males and four in females and eight were localized on the right side and one on the left side. Of the six bilateral ZACDs [Figure 1], two were in males, and four were in females. Among the 15 ZACDs, two were of the unilocular type and 13 were multilocular. Among the eight ZACDs found on the right side, two (25%) were of the unilocular type and six (75%) were of the multilocular type [Figure 2]. The only one (100%) ZACD that was found on the left side was of the multilocular type [Figure 3]. The association of ZACD with gender and laterality is depicted in Figure 4.

## Discussion

The prevalence of ZACDs in the present study was 2.5%. There was no statistical difference in the male: female ratio. The mean age of the subjects with ZACDs was 30.2 years. The incidence of ZACDs decreased with age, as found in other studies as well.[2,3,5]

There were nine unilateral ZACDs and six bilateral ZACDs in the present study. This suggests that ZACDs mostly occur unilaterally, which is consistent with previous studies.[2–5]

Among the nine unilateral ZACDS, eight occurred on the right side and only one occurred on the left side. The reason for this preference for the right side is not known. In contrast to our findings, Carter *et al*. and Park *et al.* have reported an almost equal distribution of ZACDs.[2,4] This difference may be due to variations in the sample sizes as well as the populations studied. Among the 15 ZACDs, two were unilocular and 13 were multilocular, making the multilocular type by far the more common type. Similar results were obtained in some studies,[1] though there have been other studies that have reported an almost equal number of unilocular and multilocular ZACDs.[2,5] These differences might have been because of variations in the sample sizes as well as due to inter-observer variability in determining the types of ZACDs.

To conclude, if the zygomatic process of the temporal bone or the articular eminence exhibits a unilocular or multilocular radiolucency which is incidental then it is suggestive of a ZACD. If the clinician desires to confirm the diagnosis, advanced imaging techniques such as CT scan can be conclusive. It is of utmost importance that radiologists, diagnosticians, and surgeons be aware of this entity so that precise identification can be made, which not only prevents unnecessary investigations and explorations but also forewarns the surgeons and thus helps prevent potential complications. The present study demonstrates the characteristics and variations of ZACDs among a south Indian population. Further research with increased variables could provide further insights into this little known entity.
